# Differential Angiogenic Gene Expression in *TP53* Wild-Type and Mutant Ovarian Cancer Cell Lines

**DOI:** 10.3389/fonc.2014.00163

**Published:** 2014-06-20

**Authors:** Brittany Anne Davidson, Jennifer M. Rubatt, David L. Corcoran, Deanna K. Teoh, Marcus Q. Bernardini, Lisa A. Grace, William John Soper, Andrew Berchuck, Sharareh Siamakpour-Reihani, Wei Chen, Kouros Owzar, Susan K. Murphy, Angeles Alvarez Secord

**Affiliations:** ^1^Department of Obstetrics and Gynecology, Division of Gynecologic Oncology, Duke Cancer Institute, Durham, NC, USA; ^2^Institute for Genome Sciences and Policy, Duke University Medical Center, Durham, NC, USA; ^3^Gynecology Oncology, Toronto-Sunnybrook Regional Cancer Centre, Toronto, ON, Canada; ^4^Department of Radiation Oncology, Duke University Medical Center, Durham, NC, USA; ^5^Department of Biostatistics and Bioinformatics, Duke University Medical Center, Durham, NC, USA

**Keywords:** angiogenesis, *TP53*, ovarian carcinoma, hypoxia, VEGF

## Abstract

**Objectives:** Underlying mechanisms regulating angiogenesis in ovarian cancer have not been completely elucidated. Evidence suggests that the *TP53* tumor suppressor pathway and tumor microenvironment play integral roles. We utilized microarray technology to study the interaction between *TP53* mutational status and hypoxia on angiogenic gene expression.

**Methods:** Affymetrix U133A arrays were analyzed for angiogenic gene expression in 19 ovarian cancer cell lines stratified both by *TP53* mutation status and A2780 wild-type (wt) *TP53* vs. mutated (m) *TP53* cell lines after treatment under hypoxic conditions or with ionizing radiation.

**Results:** Twenty-eight differentially expressed angiogenic genes were identified in the m*TP53* cell lines compared to wt*TP53* lines. Five genes were upregulated in m*TP53* cells: 40% involved in extracellular matrix (ECM) degradation [matrix metalloproteinase 10 (MMP10)/15] and 60% in angiogenesis (fibroblast growth factor receptor 3/VEGFA/ephrin receptor-B4). Twenty-three genes were upregulated in wt*TP53*: nearly 22% were ECM constituents or involved in ECM degradation; over 40% were growth factors or mediators of angiogenesis. Five genes were upregulated in the *A*2780m*TP53* cells: 40% involved in ECM remodeling (MMP10, ADAMTS1), 40% with pro-angiogenic activity (EFNB2, factor 2 receptor), and 20% with anti-angiogenic properties (ADAMTS1). Three genes were upregulated in hypoxia treated cells compared to controls: one with anti-angiogenic activity (angiopoietin-like 4) and two with pro-angiogenic activity (VEGFA, EFNA3). No significant gene fold changes were noted after exposure to radiation. Four genes continued to demonstrate significant differential expression (*p* ≤ 0.05) after adjusting for multiple comparisons. These genes included *endoglin* upregulation in wt lines (pro-angiogenesis) and upregulation of *FGF20* (growth factor), *ADAMTS1* (anti-angiogenesis) and *MMP10* (ECM degradation) in m*TP53* cell lines.

**Conclusion:** Our exploratory findings indicate that non-overlapping angiogenic pathways may be altered by *TP53* mutations and hypoxic conditions in the tumor microenvironment. Further evaluation is needed for confirmation.

## Introduction

The underlying mechanisms that regulate angiogenesis in ovarian cancer have yet to be elucidated but most likely involve interactions controlled by tumors and their microenvironment. Angiogenesis is a complex multistep process that includes increased vascular permeability and dilation followed by extracellular matrix (ECM) degradation; subsequent endothelial cell proliferation and migration; formation of endothelial tubes; and recruitment of pericytes to support the neovasculature. Both tumor and host tissues produce angiogenic factors that influence endothelial cell development and migration (1). The vascular endothelial growth factor (VEGF) family (VEGF-A, -B, -C, FIGF) and fibroblast growth factor 2 (FGF2) are fundamental growth factors in the process of angiogenesis with VEGF-A having the most pro-angiogenic activity. *VEGF* promoter activity, *VEGF* mRNA levels, and *FGF2* mRNA expression have been shown to be downregulated by wild-type (wt) *TP53* (2–4). In mutant (m) *TP53* it has been demonstrated that *hypoxia inducible factor 1* (*HIF1*) dependent transcriptional activation of *VEGF* gene expression has been enhanced (2). *TP53* dysfunction has also been associated with increased tumor angiogenesis based on microvessel density (MVD) by immunohistochemistry (IHC) (5, 6). These findings indicate that *TP53* may play a role in the regulation of angiogenesis in ovarian cancer.

Hypoxic conditions in the tumor microenvironment have been shown to stimulate angiogenesis in prostate (7), breast (7), melanoma (8), and renal cancer (9). As the distance from tumor to blood supply is increased, the leading edge of the tumor becomes hypoxic, and in turn, induces the expression of the *HIF1*/*hypoxia regulatory element* (*HRE*) complex, a key transcription factor. HIF1α is considered a key regulator of angiogenic factors. *HIF1/HRE* is responsible for increased gene expression of numerous genes involved in angiogenesis, cell proliferation, and matrix metabolism (10).

In order to investigate the interaction between tumor molecular biology and the microenvironment on the regulation of angiogenesis in ovarian cancer, we utilized genome-scale molecular technology. Our results can enhance our understanding of the molecular profiles of ovarian cancer tumor microenvironment and link crucial processes such as angiogenesis, hypoxia, and perfusion; all of which are established factors in tumor aggressiveness and resistance to therapy. Our primary objective was to determine if angiogenic genes are differentially expressed in ovarian cancer cell lines containing wt vs. m*TP53* genes. We also sought to investigate angiogenic gene expression patterns after simulated induction of *TP53* and hypoxia-related pathways. Our goal was to identify novel angiogenic targets that may be exploited for therapeutic purposes.

## Materials and Methods

### Ovarian cancer cell lines

Eighteen immortalized ovarian cancer cell lines maintained by the Duke Gynecologic Oncology research labs (Table 1) were sustained in monolayer culture in RPMI 1640 with 10% fetal bovine serum, sodium pyruvate, glutamine, and non-essential amino acids in 5% CO_2_ humidified chambers. Cell line authentication was performed using the AmpFlSTR^®^ Identifiler^®^ Plus PCR Amplification Kit (Applied Biosystems, Carlsbad, CA, USA) at the University of Colorado Cancer Center, DNA Sequencing, and Analysis Core (11). The STR genotypes of ovarian cancer cell lines that are available from the American Type Culture Collection or the RIKEN BioResource Center Cell Bank were identical to the source genotypes as reported within their respective STR databases and all other non-commercially available cell lines were shown to be derived from females with unique genotypes. The A2780wt*TP53* and A2780m*TP53* cell lines were obtained from Professor Robert Brown B.Sc., Ph.D., of the Department of Medical Oncology, University of Glasgow. Protein extractions were performed as previously described (12). All experiments were performed in duplicate or quadruplicate with appropriate controls.

**Table 1:** **Immortalized ovarian cancer cell lines stratified by *TP53* status**.

Wild-type *TP53* cell lines	Mutated *TP53* cell lines
A2780 parent cell line^a^	A2780 mutant cell line^a^
DOV13	Fuov1
HEY	TOV112D
HEYA8	OV90
HEYC2	OVCAR10
OVCA429	OVCAR3
TOV21G	OVCA432
	PEO1
	PEO4
	IGROV1
	OVCA420
	Tyknu
	TyknuCisR

*^a^ The A2780 cell lines (parent and mutant) were not included in the larger microarray analysis. These cell lines were evaluated separately*.

### Hypoxia and radiation treatment of cell lines

A2780 cell lines were grown to 80% confluence in T150 flasks and exposed to hypoxic conditions using 5% O_2_ in a Bactron Anaerobic Chamber (Sheldon Manufacturing, Cornelius, OH, USA) for 8 or 24 h prior to harvesting. For radiation exposure, the A2780wt*TP53* and A2780m*TP53* cell lines were plated in 60 mm dishes, exposed to 5 Gy of ionizing radiation for 900 s using the Gammacell 1000 (MDS Nordion, Ottawa, ON, Canada) and harvested at 0, 2, 4, 6, 8, 24, and 48 h. Western blots were performed to evaluate p53 and p21 protein expression per established protocols (12). The following antibodies were used: p53 (sc-126, mouse monoclonal, Santa Cruz Biotechnology, Santa Cruz, CA, USA), p21 (AB-11, mouse monoclonal, NeoMarkers, Fremont, CA, USA), actin (A4700, mouse monoclonal, SIGMA, St. Louis, MO, USA), and goat anti-mouse secondary antibody (Jackson ImmunoResearch, West Grove, PA, USA).

### Microarray sample preparation

**Figure 1:**
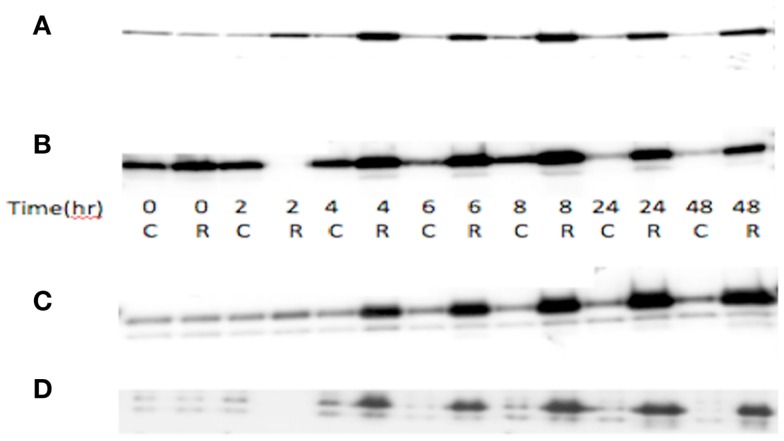
**TP53 and p21 protein expression in radiated (R) vs. control (C) in A2780 cell line**. **(A)** Wild-type A2780 cell line shows increased TP53 expression in radiated vs. control samples. **(B)** *TP53* mutant A2780 cell line with induction of TP53 expression in radiated vs. control samples. TP53 protein expression increase seen at 4 h, with greatest increase at 6–8 h, and induction sustained to 48 h. **(C)** p21 protein expression in wild-type cells. Increased expression noted at 4 h and sustained to 48 h. **(D)** p21 expression in *TP53* mutant cell line. Induction of expression noted at 4 h, sustained to 48 h.

Total RNA was used for probe generation and hybridization to Affymetrix U133A GeneChip arrays as has been described previously in detail (13, 14). The microarray data was screened to select for 378 probe sets belonging to angiogenic candidate genes on the array, based on literature review. Expression patterns were compared between: (1) wt*TP53* vs. m*TP53* ovarian cancer cell lines; (2) hypoxia treated and untreated controls using the A2780wt*TP53* and A2780m*TP53* cell lines; (3) radiated A2780wt*TP53* after 8 h of exposure and untreated controls. The 8-h sample was chosen because p53 protein expression after radiation exposure was highest between 6 and 8 h (Figure 1).

### Statistical methodologies

Microarray expression was calculated using the robust multi-array average (RMA) algorithm implemented in the *affy* package (15) of the Bioconductor (16) extensions to the R statistical programming environment (http://www.R-project.org). RMA generates a background-corrected and quantile-normalized measure of expression on the log2 scale of measurement. The ovarian cancer cell line data, and that generated from the different A2780 conditions, were each analyzed separately in this manner. For each probe set on the array, we used a moderated *T*-statistic from the *limma* package (17) to identify differential expression between the wt and m*TP53* genotypes. To identify differential expression between the wt and mutant *TP53* A2780 cell lines, hypoxia treated cells, and radiated cells, we fit a three way analysis of variance (ANOVA) model with fixed effects for genotype, treatment, and batch for each probe set on the array. Thus genotype effects are treatment and batch corrected, while treatment effects are genotype and batch corrected. The untreated A2780wt*TP53* samples served as the baseline for this analysis. The Holm–Bonferroni method was used to correct for multiple hypothesis testing. Candidate genes with an unadjusted *p*-value <0.05 and an absolute value log2 fold change (L2FC) >1.0 were identified. With these candidate genes, a two-sample *t*-test was used to analyze the gene expression data available in The Cancer Genome Atlas (TCGA) database. The Benjamini–Hochberg method was used to control the false discovery rate. Clustering of genes for heatmap presentation was done using a correlation distance metric on the *z*-score normalized expression values.

## Results

### Angiogenic-related gene expression in ovarian cancer cell lines

**Figure 2:**
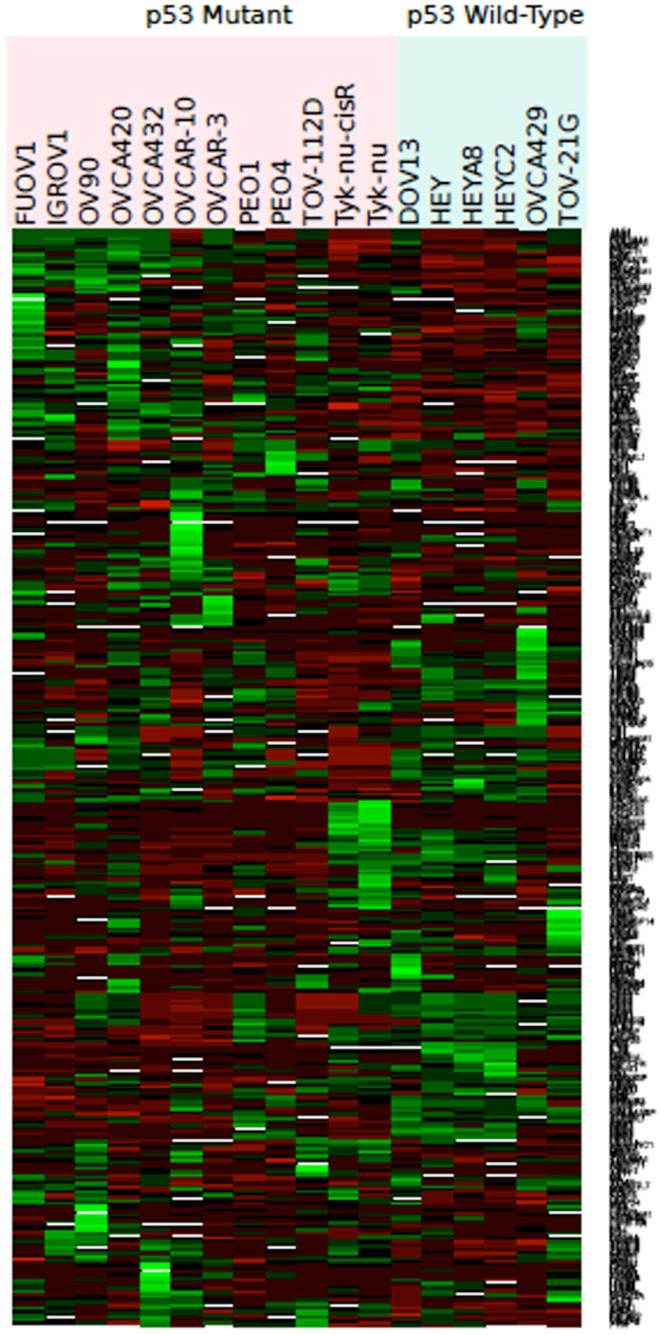
**Heat map representing color-coded expression of differentially expressed genes in 18 different wild-type or mutant *TP53* cell lines**. Twenty-eight genes exhibited statistically significant differential expression by microarray analysis based on *TP53* status.

Eighteen ovarian cancer cell lines with known *TP53* genotype (wt vs. m) were analyzed (Table 1). Of the 378 angiogenic candidate gene probes identified during literature review, 28 (7.4%) were found to be differentially expressed in cell lines with wt vs. m*TP53* genotype status (Table 2; Figure 2). Five genes (five probe sets) were considered upregulated in the m*TP53* cell lines compared to those with a wt*TP53* gene given our initial threshold of a *p*-value ≤0.05 and an absolute value L2FC ≥1. This list includes *fibroblast growth factor receptor 3* (*FGFR3*) (7.0 FC), *matrix metalloproteinase 10* (*MMP10*) (5.7 FC), *VEGFA* (3.2 FC), *MMP15* (2.5 FC), and *ephrin receptor-B4* (*EPHB4*) (2.0 FC). After correcting for multiple hypothesis testing, none of the genes were considered significant at an adjusted *p*-value <0.05.

**Table 2:** **Angiogenic genes that are differentially expressed in ovarian cancer cell lines harboring a mutant *TP53* gene compared to those with an intact wild-type *TP53* gene**.

Gene	Gene name	Function	Probe	*p*-Value	Adjusted *p*-value	Log2 fold change	Fold change
**GENES UPREGULATED IN MUTANT** ***TP53*** **CELLS LINES COMPARED TO WILD-TYPE** ***TP53*** **CELL LINES**							
FGFR3	Fibroblast growth factor receptor 3	Tyrosine kinase receptor	204379_s_at	0.007	0.10	2.8	7.0
MMP10	Matrix metallopeptidase 10	ECM degradation	205680_at	0.04	0.22	2.5	5.7
VEGFA	Vascular endothelial growth factor-A	Growth factor, angiogenesis	210512_s_at	0.008	0.10	1.7	3.2
MMP15	Matrix metallopeptidase-15	ECM degradation	203365_s_at	0.05	0.23	1.3	2.5
EPHB4	Ephrin receptor-B4	Tyrosine kinase receptor; vascular development	202894_at	0.002	0.08	1.0	2.0

**GENES UPREGULATED IN WILD-TYPE** ***TP53*** **CELLS LINES COMPARED TO MUTANT** ***TP53*** **CELL LINES**							
CTGF	Connective tissue growth factor	Mitogen secreted by endothelial cells	209101_at	0.004	0.08	3.8	14.0
SERPINE1	Serpine peptidase inhibitor, clade E, member 1	Fibrinolysis inhibition	202628_s_at	0.004	0.08	3.8	14.0
			202627_s_at	0.02	0.13	3.1	8.6
PLAU	Plasminogen activator urokinase	ECM degradation	211668_s_at	0.003	0.08	3.7	13.0
			205479_s_at	0.01	0.10	3.1	8.6
CD44	CD44 antigen	Cell surface glycoprotein	212063_at	0.014	0.13	3.5	11.3
			210916_s_at	<0.001	0.05	3.0	8.0
			212014_x_at	0.005	0.09	3.0	8.0
			209835_x_at	0.007	0.10	3.1	8.6
			204490_s_at	0.007	0.10	2.9	7.5
			217523_at	0.016	0.13	1.8	3.5
			204489_s_at	0.007	0.10	2.8	7.0
THBS1	Thrombospondin 1	Adhesive glycoprotein	201110_s_at	0.01	0.13	3.3	9.8
			201109_s_at	0.01	0.13	3.3	9.8
			201108_s_at	0.02	0.13	2.9	7.5
			215775_at	0.05	0.22	1.1	2.1
ANPEP	Alanyl aminopeptidase	Metabolism of regulatory peptides	202888_s_at	0.03	0.19	3.2	9.2
NRP1	Neuropilin 1	Multifunctional membrane receptor	212298_at	0.002	0.08	3.2	9.2
			210510_s_at	0.015	0.13	2.1	4.3
ENG	Endoglin, CD105	Endothelial cell surface protein	201809_s_at	<0.001	0.01	3.1	8.6
			201808_s_at	<0.001	0.01	1.8	3.5
TGFA	Transforming growth factor, alpha	Growth factor	205016_at	0.003	0.08	3.1	8.6
COL4A2	Collagen, type IV, alpha 2	ECM constituent	211964_at	0.05	0.22	3.0	8.0
			211966_at	0.03	0.18	2.3	4.9
COL4A1	Collagen, type IV, alpha 1	ECM constituent	211980_at	0.04	0.22	2.8	7.0
			211981_at	0.02	0.13	2.7	6.5
IL1B	Interleukin-1β	Mediator of inflammatory response	205067_at	0.002	0.08	2.8	7.0
			39402_at	0.003	0.08	2.3	4.9
FGF2	Fibroblast growth factor 2	Growth factor	204422_s_at	0.003	0.08	2.4	5.3
			204421_s_at	0.004	0.08	1.7	3.2
SPHK1	Sphingosine kinase 1	Kinase; anti-apoptotic pathways	219257_s_at	0.006	0.10	2.1	4.3
EFEMP2	EGF-containing fibulin-like extracellular matrix protein	ECM protein	206580_s_at	0.018	0.13	1.9	3.7
			209356_x_at	0.013	0.13	1.6	3.0
CXCL2	Chemokine ligand-2	Regulates hematopoietic progenitor proliferation	209774_x_at	0.05	0.22	1.9	3.7
PLAUR	Plasminogen activator, urokinase receptor	ECM degradation	211924_s_at	0.04	0.20	1.8	3.5
F2R	Coagulation factor 2 (thrombin) receptor	G-protein coupled receptor, mediates endothelial cells activation	203989_x_at	0.008	0.10	1.5	2.8
NRP2	Neuropilin 2	Multifunctional membrane receptor	211844_s_at	0.004	0.08	1.5	2.8
			219367_s_at	0.004	0.08	1.2	2.3
EPHB2	Ephrin receptor-B2	Tyrosine kinase receptor; possible tumor suppressor	209589_s_at	0.04	0.20	1.2	2.3
			211165_x_at	0.03	0.19	1.2	2.3
EDIL3	EGF-like repeats and discoidin I-like domains 3	Integrin ligand; mediates angiogenesis	207379_at	0.03	0.19	1.2	2.3
ZFP36L1	ZFP ring finger protein-line 1	Regulates response to growth factors	211962_s_at	0.02	0.13	1.1	2.1
EGFR	Epidermal growth factor receptor	Tyrosine kinase receptor; cell signaling	211607_x_at	0.03	0.20	1.1	2.1

*ECM, extracellular matrix*.

Twenty-three genes (43 probe sets) were upregulated in the wt*TP53* relative to the m*TP53* cell lines including *connective tissue growth factor* (*CTGF*) (14.0 FC), *Serpine Peptidase Inhibitor, Clade E, Type 1, Member 1* (*SERPINE1*) (14.0 FC), *plasminogen activator urokinase-type* (*PLAU*) (13.0 FC), *CD44* (11.3 FC), *thrombospondin 1* (*THBS*) (9.8 FC), *neuropilin 1* (*NRP1*) (9.2 FC), *alanyl aminoopeptidase* (*ANPEP*) (9.2 FC), *endoglin* (*ENG*) (8.6 FC), *transforming growth factor alpha* (TGF-α) (8.6 FC), *collagen type IV alpha 2* (*COL4A2*) (8.0 FC), *COL4A1* (7.0 FC), and *FGF2* (5.3 FC) (Table 2; Figure 2). Only *ENG* was significant at a *p*-value of 0.01 after correcting for multiple hypothesis testing.

### Angiogenic-related gene expression in *TP53* wt and mutant A2780 ovarian cancer cells

The A2780 cell lines differ only by a single *TP*53 gene mutation and, therefore, allowed for isolation of differences in gene expression related to this *TP*53 mutation. Five genes were upregulated in the A2780m*TP53* compared to A2780wt*TP53* cells, including *MMP10* (5.3 FC), *FGF20* (2.8 FC), *A disintegrin-like and metalloprotease with thrombospondin type 1 motif* (*ADAMTS1*) (2.8 FC), *ephrin B2* (*EPHB2*) (2.3 FC), and *coagulation F2R* (2.0 FC) (Table 3). *FGF20* (adjusted *p* < 0.001), *MMP10* (adjusted *p* = 0.004), and *ADAMTS1* (adjusted *p* < 0.001) were still considered significant after adjusting for multiple hypothesis testing. In contrast, the A2780wt*TP53* line demonstrated an upregulation in three genes: *VEGFC* (2.5 FC), *hypoxia inducible factor 1 alpha* (*HIF1A*) (2.3 FC), and *angiopoietin-like 4* (*ANGPTL4*) (2.1 FC) (Table 3).

**Table 3:** **Angiogenic genes that are differentially expressed in the A2780 wild-type and mutant *TP53* ovarian cancer cell lines and after treatment with hypoxia**.

Gene symbol	Gene name	Function	Probe	*p*-Value	Adjusted *p*-value	Log2 fold change	Fold change
**GENES UPREGULATED IN MUTANT A2780** ***TP53*** **CELLS LINES COMPARED TO WILD-TYPE A2780** ***TP53*** **CELL LINES**							
MMP10	Matrix metallopeptidase 10 (stromelysin 2)	ECM degradation	205680_at	<0.001	0.004	2.4	5.3
FGF20	Fibroblast growth factor 20	Neurotrophic factor	220394_at	<0.001	<0.001	1.5	2.8
ADAMTS1	A disintegrin and metalloproteinase with thrombospondin motif 1	Anti-angiogenic activity, ECM remodeling	222162_s_at	<0.001	<0.001	1.5	2.8
EFNB2	Ephrin B2	Endothelial cell adhesion	202668_at	0.001	0.27	1.2	2.3
F2R	Coagulation factor 2 (thrombin) receptor	G-protein coupled receptor, mediates activation of endothelial cells	203989_x_at	0.006	1	1.0	2.0

**GENES UPREGULATED IN WILD-TYPE A2780** ***TP53*** **CELLS LINES COMPARED TO MUTANT A2780** ***TP53*** **CELL LINES**							
HIF1A	Hypoxia inducible factor 1	Regulates cell response to hypoxia	200989_at	<0.001	0.09	1.2	2.3
VEGFC	Vascular endothelial growth factor-C	Growth factor, angiogenesis	209946_at	0.001	0.2	1.3	2.5
ANGPTL4	Angiopoietin-like 4	Inhibits vascular growth, tumor cell invasion	221009_s_at	0.02	1	1.1	2.1

**GENES UPREGULATED BY HYPOXIA vs. CONTROL**							
VEGFA	Vascular endothelial growth factor-A	Growth factor, angiogenesis	211527_x_at	0.002	0.9	1.6	3.0
			212171_x_at	0.003	1	1.2	2.3
			210513_s_at	0.003	1	1.4	2.6
			210512_s_at	0.001	0.4	1.8	3.5
ANGPTL4	Angiopoietin-like 4	Inhibits vascular growth, tumor cell invasion	221009_s_at	0.009	1	1.5	2.8
EFNA3	Ephrin A3	Endothelial cell migration and adhesion	210132_at	0.002	0.8	1.1	2.1

#### Hypoxia exposure

**Figure 3:**
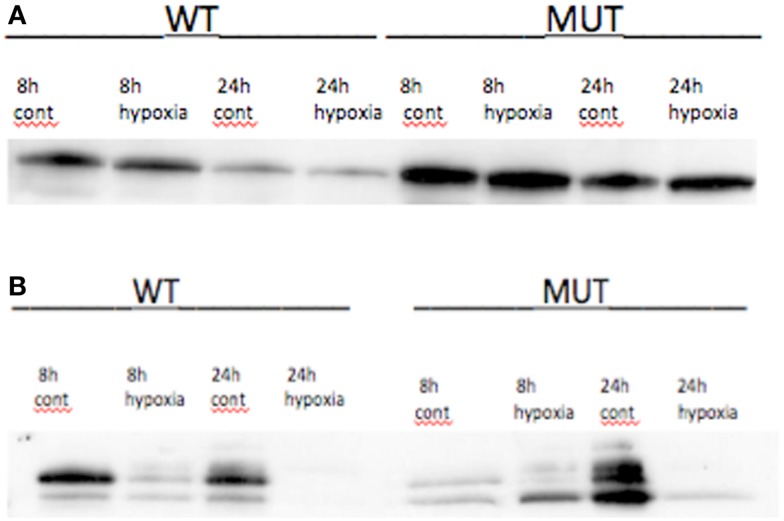
**(A)** p53 protein expression under hypoxic conditions and control in *TP53* wild-type and mutant A2780 cell lines. No changes in protein expression seen at 8 or 24 h. **(B)** Effects of hypoxia on p21 expression in A2780 cell line. In wtA2780 cell line, no induction of p21 expression was seen under hypoxic conditions. In mA2780 cell line, hypoxia induced a small increase in p21 expression at 8 h but was not present at 24 h.

**Figure 4:**
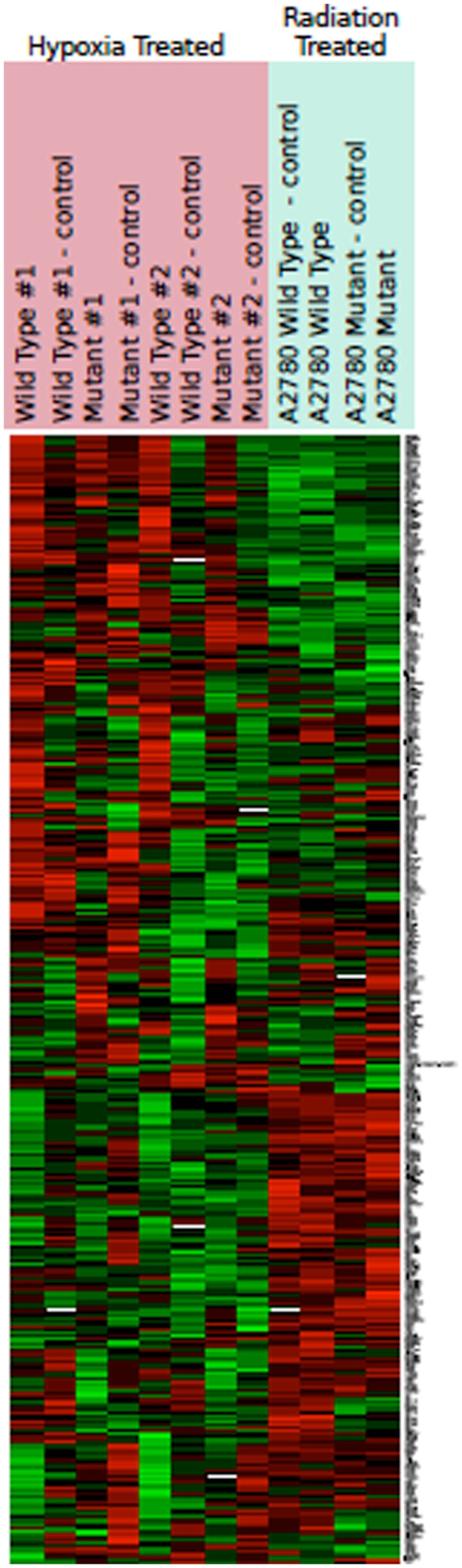
**Heat map representing color-coded expression of differentially expressed genes under hypoxic conditions and after radiation**. Numbers indicate multiple replicates performed for each sample. Three genes showed statistically significant upregulation by hypoxia. No change in gene expression was noted after radiation.

Hypoxia treatment did not increase p53 protein expression (Figure 3). A small increase in p21 protein expression was noted in the *TP53* mutant at the 8-h timepoint. This, however, was not sustained at 24 h (Figure 3). Three angiogenic genes were upregulated in hypoxia treated A2780m*TP53* cells when compared to A2780wt*TP53* controls, including *VEGFA* (3.5 FC), *ANGPTL4* (2.8 FC), and *ephrin A3* (*EPHA3*) (2.1 FC) (Table 3; Figure 4). No genes were identified as upregulated in the A2780wt*TP53* when exposed to hypoxia.

#### Radiation exposure

TP53 protein expression increase seen at 4 h, with greatest increase at 6–8 h (both mutant and wt), and induction sustained to 48 h. p21 showed a similar induction of expression, starting at 4 h and sustained to 48 h, in both wt and mutant cell lines (Figure 1). Exposure to radiation did not yield any significant differentially expressed genes when comparing the A2780m*TP53* cells to A2780wt*TP53* controls.

### TCGA database

The 36 candidate genes were then investigated using cell line data available through the TCGA database. Given that the majority of ovarian cell lines in the database had missing or discordant *TP53* status, we looked at cell lines from multiple solid tumor types. Again, after accounting for inconclusive *TP53* status and those without gene expression data, 44 cell lines were available for analysis. mRNA expression data between mutant and wt *TP53* was then compared. There was not a significant difference in expression between the two groups for any of the candidate genes, though *NRP2* (upregulated in wt; *p* = 0.06) and *MMP10* (upregulated in mutant; *p* = 0.09) trended toward significance.

## Discussion

Understanding the underlying molecular and environmentally responsive pathways driving angiogenesis can provide important insight into the regulation of tumor angiogenesis, development of resistance to VEGF-blocking agents and may assist in the identification of novel targets to exploit in the development of anti-angiogenic therapies. Our exploratory analysis indicates that the regulation of angiogenesis is complex and may be under the control of both *TP53*-dependent pathways and hypoxic conditions. Furthermore, many of the genes identified are involved in multiple facets of the angiogenic process, such as ECM degradation and remodeling; endothelial cell activation, migration, and adhesion. While our data are exploratory in nature, there were four genes that continued to demonstrate significant differential expression even after adjusting for multiple comparisons. These four differentially regulated genes included *ENG* (upregulated in wt) and *FGF20, ADAMTS1*, and *MMP10* (upregulated in mutant).

Matrix metalloproteinase 10 encodes a member of the matrix metalloproteinase family of proteins that is responsible for basement membrane degradation. MMPs are cysteine proteases with zinc ion-dependent proteolytic activity that are involved principally in the degradation of the ECM and subsequent tissue remodeling (18–20). Our findings indicate that the intact *TP53* tumor suppressor pathway may exert control via the regulation of proteins involved in the ECM. The destruction of the basement membrane and the ECM is a fundamental step in the process of tumor angiogenesis. Forty percent of the upregulated genes identified in the mutated *TP53* cell lines were involved in ECM degradation (*MMP10* and *MMP15*). MMPs are also upregulated in response to cytokines, hormone, and growth factors, including VEGF (21–23). Conversely, MMPs can also regulate the activity of various growth factors, again including VEGF (24), as well as chemokines, cytokines, and cell surface adhesion receptors. These components are involved in cell migration and intracellular communication that are directly implicated in wound healing, angiogenesis, tumor progression, and metastasis (18–20, 25, 26). The induction of MMP15 (also known as membrane-type-2 MMP) has been shown to positively correlate with ovarian tumor metastases in murine xenographs (27). MMP15 has also been postulated to have a role in anti-apoptotic pathways, though the precise mechanisms remain unknown (28). *MMP10* was also significantly upregulated in mutant A2780 *TP*53 vs. wt cell lines and marginally upregulated in the TCGA mutant *TP53* vs. wt cell lines. Furthermore, *MMP10* has been shown to be highly expressed in breast (29), prostate (30), and cervical cancer (31). Compared to epithelial ovarian cancer (EOC), *TP53* mutations in these particular malignancies are much less common (32). In a study of head and neck squamous cell malignancies, *TP53* mutations were strongly associated with MMP-9 overexpression with a subsequent increase in mean vessel density (33) while elevated p53 levels were associated with decreased MMP-2 levels in patients with invasive breast cancer (34). The notable upregulation of a number of members of the MMP family in mutated *TP53* cell lines indicates that deregulation of the *TP53* pathway may play an integral role in ECM remodeling during tumor angiogenesis.

We found that several members of the *FGF* pathway exhibited differential expression. This pathway is comprised of over 20 ligands and 4 tyrosine kinase receptors (FGFRs). Selected FGF growth factors activate the FGFRs in conjunction with heparan sulfate proteoglycans leading to the regulation of cell differentiation, angiogenesis, cell motility, invasion, and survival (35). FGF20 was recently identified and may have a potential role in tumor growth and metastasis (36). Forced expression of *FGF20* resulted in increased DNA synthesis, cellular proliferation, *in vitro* transformation, and *in vivo* tumor growth (36). There is conflicting data regarding FGF20 expression in ovarian cancer. Our data indicated that *FGF20* is expressed in ovarian cancer cell lines and was upregulated in the A2780m*TP53* ovarian cancer cells compared to the A2780wt*TP53* cells. However, in the study conducted by Jeffers et al. *FGF20* mRNA was not expressed in normal ovarian tissue or the six studied ovarian cell lines (OVCAR3, SKOV3, OVCAR4, OVCAR5, IGROV1, OVAR8) (36). In contrast, Chamorro et al. reported that FGF20 is significantly elevated in EOC cells harboring mutations in the WNT/B-catenin signaling pathway (37). *FGFR3* expression may also have an important role in cancer progression. Our data show a sevenfold increase in *FGFR3* expression in mutant *TP53* lines, consistent with Kim et al.’s finding that inhibition of FGFR3 increased target cell apoptosis and decreased resistance to targeted drug therapy (38). FGFR3 overexpression has also been correlated with shorter disease free intervals and overall survival in patients with subtypes of bladder malignancies (39).

In contrast, we found that FGF2 was upregulated in A2780 wt *TP53* (5.3 FC) compared to mutant cells. We previously reported that relative high FGF2 protein expression was associated with a significant decreased risk of disease progression and death in women with advanced ovarian cancer treated with taxane and platinum-based therapy. Upon multivariate analysis, however, the association between FGF2 and clinical outcome was no longer significant (40). In addition, we did not detect an association between FGF2 expression and *TP*53 mutation status or protein expression (40). In our prior study of FGF2, we used immunoblot technology and were unable to assess whether FGF2 is located in the stromal or cellular compartment. Our current study evaluated *FGF2* only in cell lines and does not account for stromal expression of FGF2 protein. FGF/FGFR signaling is very complex and function may vary based on the interaction of the specific FGF ligand and the FGFR variant as well as regulatory factors in the tumor and microenvironment (35). Galy et al. elegantly demonstrated that p53 protein directly repressed FGF2 mRNA translation (41). These findings highlight limitations with our microarray analysis. A single microarray analysis cannot capture transcription variances or translational protein alterations. Further exploration is needed to determine if the *TP53* pathway is involved in the coordinated regulation of FGF family members.

Our paper is the first to report a potential relationship between *TP53* mutation status and *ADAMTS1* expression in ovarian cancer. In our study, *ADAMTS1* was found to be upregulated in A2780m*TP*53 cell lines compared to wt. ADAMTS1 is active in ECM degradation and remodeling (42, 43) and has been implicated in normal ovarian follicular development (44). Collagen IV, as well as other basement membrane structural proteins, are poorly organized in ADAMTS1 null ovaries (43). ADAMTS1 may also play a role in ovarian medullary vascular development (44) and its absence leads to a delay in lymphatic development (43). Conflicting evidence exists regarding ADAMTS1 expression in malignancy. A study by Freitas et al. reported decreased ADAMTS1 expression in primary breast malignancies with forced knockdown stimulating migration and invasion of tumor cells *in vivo* (45). In contrast, others have reported significant upregulation in breast malignancies with subsequent increase in metastatic activity (46, 47). These contrasting findings may result from the auto-proteolytic cleavage of ADAMTS1 with subsequent disparate effects on tumor activity – the full length molecule displays pro-tumor properties while its cleavage products, ADAMTS-1NTF and ADAMTS-1CTF, exhibit anti-tumor activity. This cleavage process is regulated by the rates of production and degradation of heparin sulfate proteoglycans in the tumor microenvironment (48). Our findings suggest that *ADAMTS1* regulation may also be controlled via the *TP53* pathway.

In contrast to the previous candidate genes with upregulation in mutant lines, *ENG* was upregulated in the wt*TP53* cell lines. ENG (CD105) is a membrane protein overexpressed in tumor-associated endothelial cells and is a marker of proliferating endothelial cells and surrogate for tumor angiogenesis. *ENG* downregulation in human EOC lines results in decreased vascular proliferation (49). Ziebarth et al. demonstrated that forced *ENG* inhibition resulted in decreased cell viability, increased apoptosis, induced double-stranded DNA damage, and increased cisplatin sensitivity in ovarian cancer cell lines (50). In our study, *ENG* was upregulated in the wt *TP53* cell lines and was a surprising finding given the association between increased ENG staining in MCV or tumor-associated endothelial cells and advanced stage disease, suboptimal cytoreduction (51), and increased disease progression (52, 53). There are conflicting results regarding the association between ENG and survival (51, 54, 55). High ENG expression in combination with high transforming growth factor B levels prior to chemotherapy have been associated with improved overall survival (55) while others have reported either decrease survival for those with the highest levels of ENG expression (51, 53) or no relationship at all (54). Previously, we used ENG staining to determine MVD. We did not find an association between ENG MVD and *TP53* gene mutation status or protein expression (52). The difference between our two studies may be due to the disparate study design (cell lines vs. tumor tissue) and/or methodology (gene vs. protein expression; which may not correlate) as well as the amount of stromal tissue included in the tumor tissues. Our current study evaluating *ENG* in cell lines alone does not account for stromal expression of ENG protein in the microvasculature. The association between *ENG* expression and *TP53* status in cell lines suggests that ENG may be regulated by the *TP53* pathway, though other regulatory mechanisms may also exist.

Our data also demonstrated differential regulation of other well known angiogenic genes in the m*TP53* cell lines. Most notably, *VEGFA* was upregulated in the m*TP53* cell lines and under hypoxic conditions demonstrating the convergence of the VEGF pathway by both mechanisms. VEGFA is known to be one of the most potent pro-angiogenic factors. There is conflicting literature regarding *TP53* status and association with VEGF protein expression in ovarian cancer specimens. Horiuchi and colleagues reported no association between p53 and VEGF protein expression in ovarian cancers using IHC (56). Previously, we reported an association between p53 protein overexpression and low VEGF protein expression in advanced ovarian cancer specimens, but no association between VEGF and *TP53* mutation status (40). Upon further assessment, we found that the association between p53 and VEGF protein expression was limited to the ovarian cancers that contained a wt *TP53* gene and lacked p53 protein expression (40). The lack of *VEGFA* induction with ionizing radiation suggests that higher *VEGFA* expression may be associated with *TP53* mutations, but that *TP53* may not be regulating *VEGFA* expression. In contrast, the VEGF pathway may be primarily regulated via hypoxic conditions in the tumor microenvironment rather than by *TP53*. It is well established in the literature that hypoxic conditions increase *VEGF* expression (5, 10, 56, 57) with expression increasing after exposure to hypoxia regardless of *TP53* pathway status. This suggests that hypoxia has a dominant role in *VEGF* regulation (57).

Furthermore, other members of the VEGF family, such as *VEGFC* and *NRP1* and *NRP2*, were all upregulated in the cell lines harboring wt*TP*53 compared to those with a mutant gene. *NRP2* was also marginally upregulated in the TCGA wt *TP53* cell lines compared to mutant cell lines. VEGFC is an integral part of lymphangiogenesis and has been associated with lymph node metastases and prognosis in a variety of malignancies (58–61). NRP1 and NRP2 interact with both VEGF ligands and class 3 semaphorin (SEMA3) ligands in overlapping binding domains (62). While VEGF promotes angiogenesis and interacts with NRP1 to enhance the binding to its receptor, members of the SEMA3 family inhibit angiogenesis (63). Specifically, SEMA3F binding to NRP2 inhibits tumor angiogenesis and metastasis (63). *NRP* overexpression has been reported in multiple solid malignancies including breast, gastrointestinal, and prostate tumors (63). Conflicting evidence exists regarding NRP expression in ovarian tumors. Bednarek et al. studied NRP1 expression in 50 patients with EOC, the majority of which had weak (*n* = 13) or no (*n* = 22) immunohistochemical staining (64). In contrast, Baba et al. reported that 97% of EOC tissue samples and 67% of cell lines stain strongly (65). Recently, Stanton and colleagues reported that the VEGFC–NRP2 axis promoted autophagy, which, in cancer, may represent an adaptive response to promote cell survival (62).

Members of the ephrin family were also differentially expressed in the cell lines and after exposure to hypoxia. The genes encoding the ligand EFNB2 and the receptor EPHB4 were upregulated in mutant *TP53* cells; *EFNB2* was upregulated in the A2780 line and *EPHB4* was upregulated in the larger pool of mutant *TP53* lines. In contrast, the ephrin receptor, EPHB2, was upregulated in wt *TP53* cell lines while overexpression of the ligand, EFNA3, was induced by hypoxia. Ephrin and the ephrin receptors are a family of membrane-bound tyrosine kinases and receptor tyrosine kinases (RTKs) that are typically highly promiscuous; most of the receptors are capable of binding to numerous ephrin ligands (66, 67). Since both receptors and ligands are membrane-bound, the receptor ligand interactions are capable of bi-directional signaling (67). Members of the ephrin RTK family are expressed on both tumor cells as well as the tumor endothelium and fibroblasts (68, 69). Data from targeted disruption of Eph RTKs and ligands in mice have revealed that the ephrin pathway plays a critical role in embryologic vascular development and tumor angiogenesis (70). EFNA1 and EFNB2 are regulated by *TP53* as well as other members of the *TP53* family, including *p73* and *p63* (71, 72). Hypoxia upregulates both mRNA and protein expression of *EPHB4, EFNB2, EPHA2*, and *EFNA1* (73). *EPHB4* RTK is expressed in 86% of invasive ovarian cancers and was associated with advanced stage, worse survival (74), and decreased response to chemotherapy (66). *EFNB1*, an alternate ligand of *EPHB4*, has been associated with increased MVD in EOC (75). The precise mechanism of ephrin-mediated angiogenesis is unknown, but our data indicate that both the *TP53* tumor suppressor pathway and hypoxia may effect ephrin family members.

In addition, we have evaluated our panel of angiogenic genes in women with advanced, high grade serous ovarian carcinoma whose tumors had undergone microarray analysis. We conducted an extreme phenotype study that included women with long survival (>7 years) vs. short survival (<3 years) (76). Thirty-one genes were significantly associated with clinical outcome including several of the genes reported in our current study (*CD44−, EPHB2, HIF1A, NRP1*, and *TGFA*). Of these, high CD44 was associated with longer survival in the TCGA database. In contrast, high expression of EPHB2 and NRP1 were associated with shorter survival in an external database (76). Microarray analysis of cell lines may potentially identify genes that have prognostic significance for survival.

Limitations of our study include the use of a simple model of ionizing radiation to simulate *TP53* induction (77, 78). To validate our model, we irradiated ovarian cancer cell line OVCA420 to 5 Gy and then subjected cell lysates to immunoblot to assess total p53 expression. When compared to non-irradiated cells, irradiated cells demonstrated a 3.3-fold increase in p53 protein expression 48 h after exposure (Figure 1). We acknowledge that ionizing radiation likely induces other genes in addition to *TP53* and these unidentified genes may also play a role in angiogenesis. The *in vitro* nature of this investigation limits the application of these results to more complex systems such as living organisms. Specifically, cell lines lack the adjacent stroma that is integral to evaluate mesenchymal remodeling, and tumor angiogenesis was not assessed in this model. Another limitation of our study is that while a number of genes showed a significant change in expression, most were no longer significant after correcting the *p*-values for multiple hypothesis testing. Thus, further verification by another methodology is necessary to establish that these genes are in fact differentially expressed and not the result of statistical error. Our study incorporates data from cell lines of various epithelial ovarian histologies. Though it is well known that an overwhelming majority of serous type tumors possess *TP53* mutations, a study by the Gynecologic Oncology Group suggests p53 overexpression may be seen in one-third of mucinous or clear cell histologies while over 70% of tumors deemed “other” also possess p53 overexpression (79).

Despite these limitations the data generated from this study confirmed the complexity of angiogenesis regulation and the presence of convergent and divergent pathways controlled via *TP53*-dependent and independent mechanisms representing how genetic mechanisms and environmental conditions interact to promote a pro-angiogenic environment. Identification of multiple members of the VEGF, FGF, MMP, and ephrin families as well as other novel genes in our series indicates the existence of multiple regulatory mechanisms involved in tumor angiogenesis. The most differentially expressed genes in our panel represent appealing therapeutic targets that may be exploited to develop anti-angiogenic therapies.

## Conflict of Interest Statement

The authors declare that the research was conducted in the absence of any commercial or financial relationships that could be construed as a potential conflict of interest.
